# A Reliable Enantioselective Route to Mono-Protected N1-Cbz Piperazic Acid Building Block

**DOI:** 10.3390/molecules25245939

**Published:** 2020-12-15

**Authors:** Evanthia Papadaki, Dimitris Georgiadis, Michail Tsakos

**Affiliations:** Laboratory of Organic Chemistry, Department of Chemistry, National and Kapodistrian University of Athens, Panepistimiopolis, 15771 Athens, Greece; evapap@chem.uoa.gr (E.P.); dgeorgia@chem.uoa.gr (D.G.)

**Keywords:** natural products, piperazate, nonproteinogenic, amino acid, peptides, peptidomimetics, bioactive peptides

## Abstract

The chiral N1-Cbz, N2-*H* derivative of the piperazic acid monomer is a valuable building block in the total synthesis of natural products, comprising this nonproteinogenic amino acid. In that context, we wish to report an improved synthetic protocol for the synthesis of both (3*R*)- and (3*S*)-piperazic acids bearing the carboxybenzyl protecting group (Cbz) selectively at the N1 position. Our method builds on previously reported protocols, circumventing their potential shortcomings, and optimizing the ultimate selective deprotection at the N2 position, thus, offering an efficient and reproducible pathway to suitably modified piperazates in high optical purity.

## 1. Introduction

**Figure 1:**
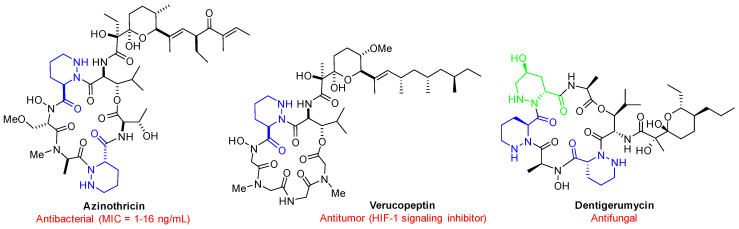
Examples of bioactive secondary metabolites with at least one piperazic acid residue embedded within their macrocyclic peptidyl core. Piz is depicted in blue color and 5-hydroxy-Piz in green.

Piperazic acid or piperazate (Piz) is a nonproteinogenic amino acid which possesses a handful of rare characteristics [1]. For example, it is the only known amino acid featuring a cyclic hydrazine motif, while its six-membered ring imparts rigidity to the parent peptide. Therefore, for instance, it can be used as a proline mimic in efforts to force β-turns in peptide chains [2]. Consequently, compounds that incorporate the Piz residue in their scaffold display a broad range of biological profiles that span from anticancer to antibiotic to antifungal and more (Figure 1) [3].

To date, a continuously growing number of secondary metabolites composed of at least one piperazic acid residue have been isolated, as well as, its post-translationally modified congeners dehydro-Piz, OH-Piz (colored green in Figure 1), and Cl-Piz [4]. Given that the vast majority of these natural products have a potent biological activity (usually going hand in hand with high cytotoxicity) and could therefore be regarded as medicinal chemistry leads, it is not surprising that many of them have been targeted for chemical synthesis, in order to fuel biological studies in a drug development or chemical biology context [3,5,6,7,8,9].

**Scheme 1:**
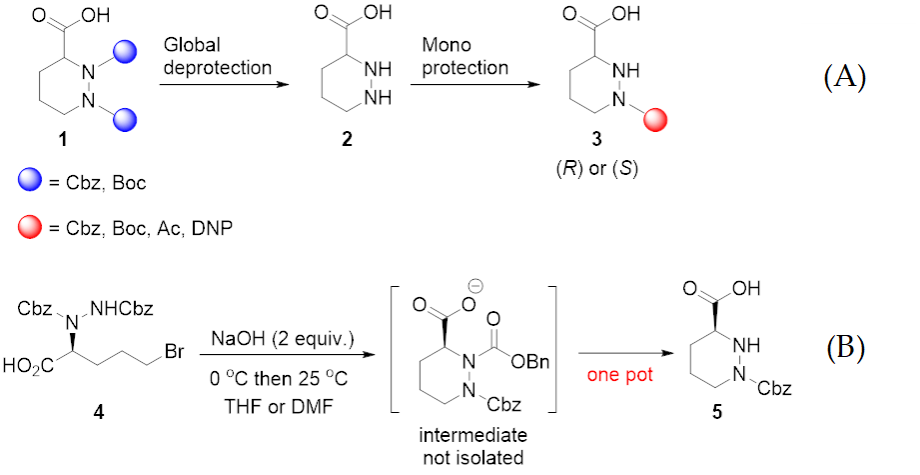
(**A**) Two-step global deprotection and re-protection of N1 position. (**B**) One-step selective deprotection of N2 position.

Barring the total synthesis of L-156,373, wherein Del Valle et al. opted for a late stage ring closure at the N-N bond [7], in all the other total synthesis projects referenced above, initially the piperazic acid monomer is prepared as decorated with the appropriate protecting groups, and then it is coupled to the adjacent amino acids to build the peptidyl portion of the natural product. However, since almost all Piz containing peptides are acylated at the N2 position, but the intrinsic propensity of the free amino acid is to react at N1, the preparation of the orthogonally mono-protected N1-carbamate Piz building block has been challenging and lengthy [2,10]. To date, the method of choice for preparing both enantiomers of N1-protected N2-*H* Piz (**3**, Scheme 1) includes a three-step sequence to form the hexahydropyridazine derivative **1** bearing two identical protecting groups, followed by a global deprotection reaction and then, carefully, a selective re-protection of N1 (Scheme 1A) [11,12,13]. On the other hand, Dawei Ma et al. disclosed recently an expedient two-step synthesis (vide infra) of (*S*)-1-Cbz-hexahydropyridazine-3-carboxylic acid (**5**, Scheme 1) on the kilogram scale by taking advantage of one pot operations without intermediate purifications and finally a novel selective deprotection of the urethane at N2 (Scheme 1B) [14].

Our interest in the synthesis of chiral piperazates stems from undertaking a total synthesis campaign of a bioactive natural peptide comprising two Piz residues, the absolute configuration of which has yet to be determined. As usual in such circumstances, a straightforward and more important reproducible synthetic route to all the small building blocks is key to reach the aspired target, especially the building blocks known in the literature. To that end, we followed the reported protocols depicted in Scheme 1 to attain both Piz enantiomers on a gram scale, only to discover that even though both strategies displayed considerable advantages, such as the practicality of the former or the brevity of the latter, unfortunately they were accompanied by limitations that stymied the progress of our research. Herein, we wish to outline the problems we encountered when we attempted to replicate the procedures above, and provide a reliable pathway to chiral N1-Cbz mono-protected piperazic acids by combining bits and pieces of the literature along with a new optimization study of the ultimate step, the selective cleavage of the Cbz group at N2.

## 2. Results and Discussion

**Scheme 2:**
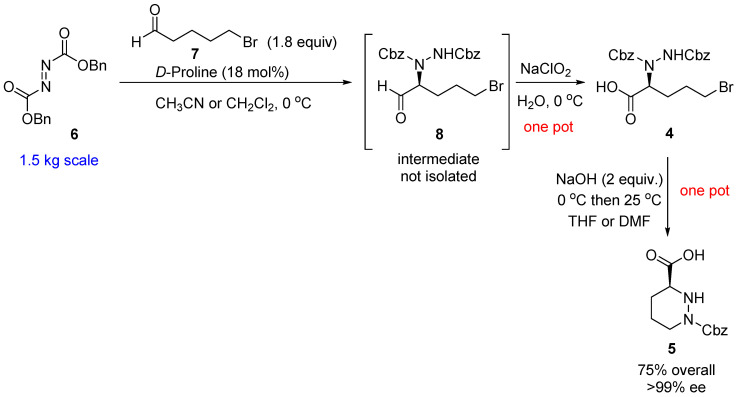
The synthetic protocol to **5** reported by Ma et al. [14].

Allured by its simplicity and step economy, we initially decided to follow the Dawei Ma protocol for the synthesis of our target building block **5** [14]. In their work, the Ma group starts with an organocatalytic asymmetric α-hydrazination reaction, first reported by List and then adjusted by the Hamada group [15,16], to obtain aldehyde **8** which is then oxidized in situ to carboxylic acid **4** (Scheme 2). The treatment of **4** with an excess of base at 0 °C forms the 6-membered ring which, upon heating to ambient temperature undergoes a series of intramolecular cascade reactions to furnish product **5** in a 75% overall yield and >99% enantiomeric excess (Scheme 2). Since the reactions were performed on the kilogram scale, both purifications involved the precipitation of the product as a white solid.

**Scheme 3:**
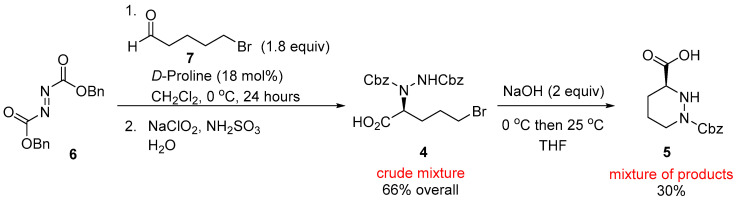
Our efforts to procure N1-Cbz-Piz by adopting Ma’s protocol.

We commenced by synthesizing bromo-aldehyde **7** in two steps (see SI) from the commercial 1,5-pentanediol [17], and then we emulated the conditions for the proline-catalyzed asymmetric α-hydrazination, as described by the Ma group (Scheme 3). Unfortunately, although ostensibly the first organocatalytic step proceeded smoothly, the subsequent Pinnick oxidation, in our hands, generated a crude mixture that could not be purified by extractions nor precipitation as stated in Ma’s protocol. Additionally, we were able to purify acid **4** by column chromatography, but not without taking a heavy toll on the yield (40–66%). We repeated the reaction several times on a scale ranging from 0.2 to 1.3 grams, and every time we failed to precipitate and filter out the clean acid. Nonetheless, we continued with crude acid **4** to the next step, which included the treatment with 2 equivalents of NaOH at 0 °C for 24 h and then at 25 °C for 4 h, as reported. However, to our dismay, we obtained after extractions a crude mixture of mono- and bis-Cbz protected Piz (approx. 30%), along with unidentified impurities that we probably carried from the previous step. All attempts to purify the product by precipitation proved futile on that scale. We attribute these discrepancies with the literature to the vast difference of the scale that the reactions were run on. Process chemistry has taught us that a reaction that works well on a milligram scale may prove to be inefficient at a much larger scale. Accordingly, it is only safe to assume that vice versa, scaling down from kilograms to milligrams may have a somewhat similar impact on the reaction’s performance, in addition to purifications, such as precipitation or crystallization, becoming much more challenging.

**Scheme 4:**
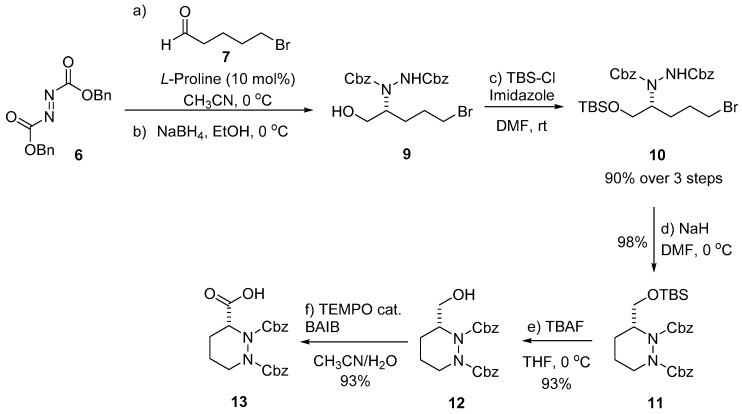
Synthesis of (*R*)-bis-Cbz-Piz **13** following the Hamada method [16].

Once we fell short of reproducing Ma’s protocol, we turned our attention to the longer but more tried route of the Hamada group [16]. In their article, Hamada et al. contend that their method stands out due to its simplicity and high yields, features that we can indeed corroborate as all reactions on the scale of 1 to 5 grams proceeded as reported and were nearly quantitative (Scheme 4). As previously, we began with the organocatalytic asymmetric α-hydrazination, only this time it was followed by the reduction of the aldehyde to the bromo-alcohol **9,** which was protected as a silyl ether to give **10** in an excellent yield of 90% and >99% *er* after three transformations. Then, a base induced intramolecular S_N_2 displacement of the bromide furnished tetrahydropyridazine **11** quantitatively. Silyl ether deprotection and TEMPO-catalyzed oxidation of the primary alcohol to the corresponding acid completed the synthesis of the (*R*)-bis-Cbz-Piz derivative **13** in high yield (overall yield 76% for six transformations) and optical purity, as depicted in Scheme 4.

**Scheme 5:**
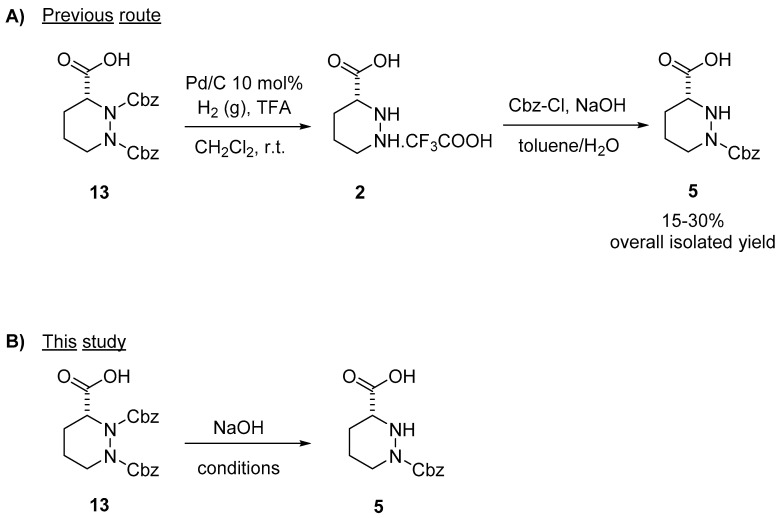
(**A**) Previous two-step formation of the mono-protected Piz building block **5**. (**B**) Selective deprotection of **13** under basic conditions to furnish **5** in one step.

With the key intermediate **13** in hand, we continued with the most practiced sequence of cleaving both Cbz-protecting groups simultaneously and then re-protecting the N1 position, as mentioned in the introduction (Scheme 1A). Therefore, we applied standard hydrogenolysis conditions (10% Pd/C under a hydrogen atmosphere at ambient temperature) in the presence of 10 equivalents of trifluoroacetic acid to obtain the protecting group-free Piz (*R*)-**2** as its trifluoroacetic salt [16], followed by the treatment with exactly one equivalent of benzyl chloroformate at low temperature to furnish the desired mono-Cbz protected derivative (*R*)-**5** (Scheme 5A) [18]. Unfortunately, once more, we experienced low overall yields and a reaction sequence that behaved erratically and was lacking reproducibility. In the first deprotection step, the product did not crystallize and we ended up with a crude slurry oil, while in the second step, we always obtained mixtures of mono- and bis-Cbz protected Piz in different proportions each time. Issues with this sequence have also been reported by others, for instance, the Hamada group observed aromatization of the Piz ring during hydrogenolysis in protic solvents [16], while other groups also disclose low overall yields due to “undetermined causes” [6,19,20].

Disappointed yet again by the outcome, but at the same time fully contented by the efficiency of the current route to bis-protected Piz building block **13**, we contemplated whether this route could converge with Ma’s selective deprotection strategy to give rise to **5** in a facile and reliable fashion (Scheme 5B). Based on the reaction’s mechanism postulated by Ma et al., one equivalent of base would have sufficed to induce the selective cleavage of the Cbz group at N2 [14]. To that end, we treated Piz derivative **13** with 1.2 equivalents of NaOH in THF at ambient temperature and we monitored the reaction by TLC analysis (entry 1, Table 1). To our delight, we observed the formation of the product after 2–3 h. However, in stark contrast to Ma’s findings where 3 h were ample time for a complete deprotection, we obtained a mixture of starting material (mostly) and product even after 18 h of stirring. Nevertheless, since our initial attempt was deemed promising, we decided to undertake an optimization study for that last deprotection step, results of which are depicted in Table 1 (for the full optimization study see Table S1 in the Supporting Information). At this point, we should mention that the reactions were monitored by TLC analysis and were evaluated as pass/fail based on the disappearance of the starting material or not. Reactions where compound **13** had been fully consumed were purified by extracting the product from the aqueous phase after adjusting the pH to 4–5, without the need for column chromatography (Table 1). Therefore, we first increased the amount of NaOH to 2 equivalents (entry 2, Table 1), which led to a slightly better ratio of product over starting, but still there was no full consumption (as judged by the TLC analysis). Then, we tested different bases (entries 3-5, Table 1), as well as, different solvents (entry 6, Table 1) without witnessing any improvement. Finally, it was not until we raised the reaction’s temperature that we observed almost complete fading of the reactant’s spot on the TLC plate after 18 h (entry 7, Table 1). A result that we have since reproduced numerous times with the isolated yield of clean **5** ranging from 60–72% due to the solubility of amino acid **5** also in the aqueous phase. Of note, when we increased 20-fold the scale of the reaction we observed the same result (entry 8, Table 1), which for our intents and purposes constitutes a proof of concept. The enantiomeric ratio of building block **5** was measured 94:6 by chiral HPLC after conversion to the respective allyl ester (see SI), a result that indicates a small drop in enantioselectivity due to the harsher conditions employed (45 °C, 18 h).

## 3. Materials and Methods 

All the reactions with air and moisture sensitive compounds were conducted in a flame dried glassware under an atmosphere of argon. All solvents, anhydrous or not, were used as purchased. If not otherwise stated, chemical reagents were used as received from commercial suppliers. The TLC analysis was carried out on silica coated aluminum foil plates (Merck Kieselgel 60 F_254_). The TLC plates were visualized by UV irradiation and/or by staining with the phosphomolybdic acid (PMA), cerium ammonium molybdate (CAM), or KMnO_4_/EtOH stain. Flash column chromatography was carried out using silica gel (230–400 mesh particle size, 60 Å pore size) as the stationary phase. The optical rotation was measured on a Perkin Elmer 343 polarimeter. Electron spray ionization (ESI) mass spectra were recorded on a Finnigan, Surveyor MSQ Plus spectrometer. ^1^H and ^13^C spectra were recorded on a Bruker Avance Neo (400 and 100 MHz, respectively) or a Varian Mercury (200 and 50 MHz, respectively). Chemical shifts (*δ*) are reported in ppm relative to the residual solvent signals. Multiplicities are indicated using the following abbreviations: s = singlet, d = doublet, t = triplet, q = quartet, m = multiplet, br = broad. High performance liquid chromatography (HPLC) was used to determine the enantiomeric excess and was performed on an Agilent 1100 Series apparatus using Chiralpak ® AD-H and OD-H as the chiral columns.

## 4. Conclusions

When we set out for our total synthesis project we did not anticipate that it would take us more than 1 year and a tremendous amount of energy to standardize the production of the piperazic acids and their derivatives. Specifically, for the syntheses of (*S*)- and (*R*)-N1-Cbz-Piz (**5**), a number of routes are currently available. However, the fact that every time that last sequence of double deprotection and selective re-protection required fine tuning, should have presaged the difficulties involved in this endeavor. In conclusion, we report on a synthetic route for the facile synthesis of enantiomerically enriched N1-Cbz piperazic acids that we believe complements the current literature, and is consistent on a small to medium scale. For a large scale production (multi decagram or kilogram), we do recommend following Ma’s protocol in which the precipitation of the products is reported to be feasible and straightforward. Since the piperazic acid residue constitutes a bona fide pharmacophore and nowadays more and more bioactive piperazyl molecules are brought into the limelight, we aspire that the synthetic course described herein will become the benchmark protocol for the production of the most valuable building block. 

## Figures and Tables

**Table 1:**
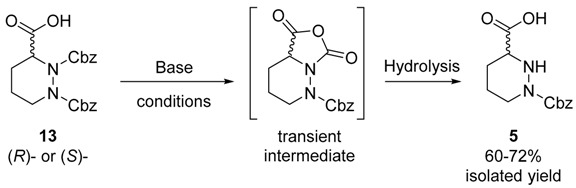
Optimization of the selective mono-deprotection reaction.

Entry ^a^	Base (equiv.)	Solvent	Temperature (°C)	Full Consumption of 13 ^b^
1	NaOH (1.2)	THF	23	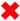
2	NaOH (2.0)	THF	23	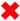
3	KOH (2.0)	THF	23	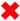
4	LiOH (2.0)	THF	23	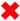
5	NaH (1.1)	THF	23	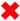
6	NaOH (2.0)	MeOH	23	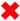
7	NaOH (2.0)	THF	45	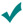
8 ^c^	NaOH (2.0)	THF	45	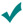

^a^ All the reactions were carried out in a screw cap vial using acid (*R*)- or (*S*)-**13** (50 mg, 0.13 mmol) in the solvent (0.26 M) at a given temperature for 18 h. ^b^ Based on the disappearance of the starting material or not by TLC. In entries 7 and 8, where full consumption was observed, the isolated yields of **5** after extractions were 72% and 60%, respectively. ^c^ The reaction was performed using 1 g (2.60 mmol) of acid **13**.

## Supplementary Materials

The following are available online. Table S1: Full optimization study of the selective deprotection reaction. Figure S1 (**Α**): Monitoring the reactions by TLC analysis. In lanes 2 and 4 where the sm is fully consumed the product was purified. Correlation between TLC lanes and Table S1: Lane 1 → Entry 7, Lane 2 → Entry 26, Lane 3 → Entry 15, Lane 4 → Entry 25, Lane 5 → Entry 23, Lane 6 → Entry 24, Lane S → starting material 13. (**B**): Reaction setup in a sand bath at 45 °C. (**C**): Reaction setup at ambient temperature; Figure S2 (**A**), (**B**), and (**C**): TLC analysis of the organocatalytic reaction sequence by Ma and co-workers; The experimental procedures and characterization data of all synthesized compounds; Images of NMR spectra; Chiral HPLC for the determination of enantiomeric excess.
