# Supplementary material for: A Reliable Enantioselective Route to Mono-Protected N1-Cbz Piperazic Acid Building Block
Source: Molecules. 2020 Dec 15;25(24):5939. doi: 10.3390/molecules25245939 (PMC7765410; doi:10.3390/molecules25245939)
Supplement: Supplementary file 1 [file molecules-25-05939-s001.pdf]

molecules

MDPI

Article

A Reliable Enantioselective Route to Mono-Protected N1-Cbz Piperazic Acid Building Block

Evanthia Papadaki, Dimitris Georgiadis and Michail Tsakos *

Laboratory of Organic Chemistry, Department of Chemistry, National and Kapodistrian University of Athens, Panepistimiopolis Athens 15771, Greece; evapap@chem.uoa.gr (E.P.); dgeorgia@chem.uoa.gr (D.G.)

* Correspondence: mitsak@chem.uoa.gr; Tel.: +30-210-727-4899

Optimization of the mono-deprotection reaction.

Table S1. Full optimization study of the selective deprotection reaction.

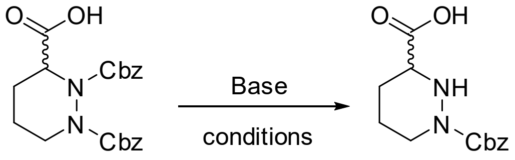

13 **5**

Entry.	Starting material	Base (equiv)	Additives (equiv)	Solvent (0.26M)	Temperature (°C)	Time (h)	Full consumption of 13 (isolated yield of 5)
1	0.13mmol (50mg)	NaOH (1.2)	-	THF	23	1	-
2	0.13mmol (50mg)	NaOH (1.2)	-	THF	23	2	-
3	0.13mmol (50mg)	NaOH (1.2)	-	THF	23	$2^{1/2}$	-
4	0.13mmol (50mg)	NaOH (1.2)	-	THF	23	18	-
5	0.13mmol (50mg)	NaOH (2.0)	-	THF	23	1	-
6	0.13mmol (50mg)	NaOH (2.0)	-	THF	23	2	-
7	0.13mmol (50mg)	NaOH (2.0)	-	THF	23	$2^{1/2}$	-
8	0.13mmol (50mg)	NaOH (2.0)	-	THF	23	18	-
9	0.13mmol (50mg)	KOH (1.2)	-	THF	23	1	-
10	0.13mmol (50mg)	KOH (1.2)	-	THF	23	2	-
11	0.13mmol (50mg)	KOH (1.2)	-	THF	23	$2^{1/2}$	-
12	0.13mmol (50mg)	KOH (1.2)	-	THF	23	18	-
13	0.13mmol (50mg)	KOH (2.0)	-	THF	23	1	-
14	0.13mmol (50mg)	KOH (2.0)	-	THF	23	2	-
15	0.13mmol	KOH	-	THF	23	$2^{1/2}$	-

	(50mg)	(2.0)					
16	0.13mmol (50mg)	KOH (2.0)	-	THF	23	18	-
17	0.13mmol (50mg)	KOH (2.0)	-	MeOH	23	1	-
18	0.13mmol (50mg)	KOH (2.0)	-	MeOH	23	2	-
19	0.13mmol (50mg)	KOH (2.0)	-	MeOH	23	$2^{1/2}$	-
20	0.13mmol (50mg)	KOH (2.0)	-	MeOH	23	18	-
21	0.13mmol (50mg)	NaOH (2.0)	-	MeOH	23	1	-
22	0.13mmol (50mg)	NaOH (2.0)	-	MeOH	23	2	-
23	0.13mmol (50mg)	NaOH (2.0)	-	MeOH	23	$2^{1/2}$	-
24	0.13mmol (50mg)	NaOH (2.0)	-	MeOH	23	18	-
25	0.13mmol (50mg)	KOH (2.0)	-	THF	45	18	✓ (45%)
26	0.13mmol (50mg)	NaOH (2.0)	-	THF	45	18	✓ (72%)
27	0.13mmol (50mg)	NaOH (3.0)	-	THF	45	18	✓ (60%)
28	0.13mmol (50mg)	NaOH (3.0)	-	THF	23	18	-
29	0.13mmol (50mg)	Rb_2CO_3 (2.0)	-	THF	45	18	-
30	0.13mmol (50mg)	Rb_2CO_3 (3.0)	-	THF	45	18	-
31	0.13mmol (50mg)	LiOH (2.0)	-	THF	23	18	-
32	0.13mmol (50mg)	LiOH (2.0)	-	THF	45	18	-
33	0.13mmol (50mg)	NaH (1.1)	-	THF	0 to 23	2	-
34	0.13mmol (50mg)	NaH (1.1)	-	THF	0 to 23	18	-
35	0.13mmol (50mg)	NaOH (2.0)	-	THF	23	18	-
36	0.13mmol (50mg)	NaOH (2.0)	Crown ether (1.1)	THF	23	18	-
37	0.13mmol (50mg)	NaOH (2.0)	Crown ether (1.1)	THF	45	18	- (35%)
38	0.13mmol (50mg)	KOH (2.0)	Crown ether (1.1)	THF	23	18	-
39	0.13mmol (50mg)	KOH (2.0)	Crown ether (1.1)	THF	45	18	- 30%
40	0.26mmol (100mg)	NaOH (2.0)	-	THF	45	18	✓ (61%)
41	1.30mmol (500 mg)	NaOH (2.0)	-	THF	45	18	✓ (70%)
42	2.6mmol (1000mg)	NaOH (2.0)	-	THF	45	18	✓ (60%)

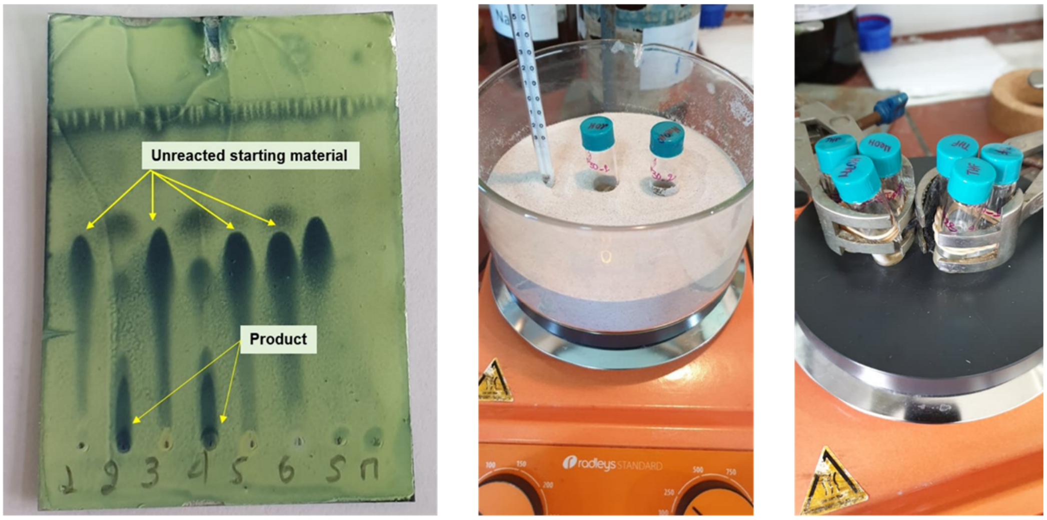

(A) (B) (C)

Figure S1. (**A)**: Monitoring the reactions by TLC analysis. In lanes 2 and 4 where the sm is fully consumed the product was purified. Correlation between TLC lanes and Table S1: Lane 1 → Entry 7, Lane 2 → Entry 26, Lane 3 → Entry 15, Lane 4 → Entry 25, Lane 5 → Entry 23, Lane 6 → Entry 24, Lane S → starting material **13**. (**B**): Reaction setup in a sand bath at 45°C. (**C**): Reaction setup at ambient temperature.

Synthetic protocols and characterization data

1-((Benzyloxy)carbonyl)hexahydropyridazine-3-carboxylic acid (5).

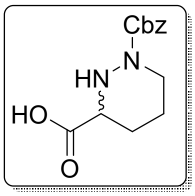

In a screw cap vial containing **13** (50 mg, 0.13 mmol, 1 equiv.) in THF (0.5 mL) was added solid NaOH (10 mg, 0.26 mmol, 2 equiv.) and the mixture was heated to 45 °C and left stirring for 18 h. After cooling to room temperature, the solvent was evaporated and the residue was dissolved in H_2O (5 mL), transferred to a separatory funnel and washed with Et_2O (5 mL). The organic layer was discarded to remove the byproduct [Figure S1(A), upper spot on TLC] and the aqueous phase was acidified with HCl 1N to pH 4-5 and then extracted thoroughly with ethyl acetate (4 x 5 mL). The combined organic layers were washed with brine, dried (Na_2SO_4), filtered and concentrated in vacuo to afford piperazic acid **5** (25 mg, 72%) as a white solid.
R_f 0.2 (9:1 chloroform/methanol, PMA stain). mp 158-160 °C, (Lit.[3]mp 166-167 °C). For (*R*)-**5** we found: $[\alpha]_D^{23}$ = +22 (*c* 1, MeOH), {[Lit.[3]$[\alpha]_D^{20}$ = -35 (*c* 1, MeOH) for (*S*)-enantiomer};^{1}H-NMR (400 MHz, CD_3OD) *δ* 7.39-7.29(br, 5H), 5.16 (br s, 2H), 3.85 (br, 1H), 3.51-3.48 (m, 1H), 3.35 (br, 1H), 2.01 (br, 1H), 1.79-1.60 (br, 3H);^{13}C NMR (101 MHz, CD_3OD) *δ* 174.7, 157.5, 137.9, 129.5, 129.3, 129.2, 129.0, 128.3, 127.99, 68.7, 65.2, 59.3, 45.7, 28.4, 24.2. MS (ESI) *m/z* (%) :265.2 $[M+H, (100)]^+$.

Synthesis of aldehyde 7.

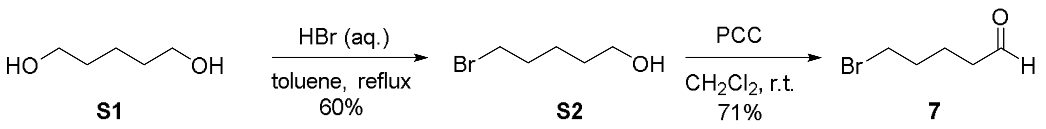

To a stirring solution of 1,5-pentanediol (2 g, 19.20 mmol, 1 equiv.) in toluene (58 mL) was added HBr (48% aq., 2.6 mL, 23.04 mmol, 1.2 equiv.) and the mixture was heated at reflux for 24 hours. After cooling down to ambient temperature, the mixture was transferred to a separatory funnel and the layers were separated. The organic phase was washed with 1N NaOH (aq., 15 mL) and brine (30 mL), dried (Na_2SO_4), filtered and concentrated carefully at the rotavap (ATTENTION: alcohol **S2** is volatile). The crude product was purified by FCC (pentane/Et_2O 1:1 to 1:1.5) to furnish **S2** (1.91 g, 60%) as a colorless liquid. NMR data matched those in the literature.[1]
R_f 0.3 (7:3 petroleum ether 40-60 °C/EtOAc, CAM stain). ^{1}H-NMR (200 MHz, $CDCl_3$) *δ* 3.66 (t, *J* = 6.2 Hz, 2H), 3.42 (t, *J* = 6.7 Hz, 2H), 1.97-1.83 (m, 2H), 1.67-1.43 (m, 4H).

To a stirring mixture of PCC (2.94 g, 13.65 mmol, 1.2 equiv.) and Florisil (11 g) in CH_2Cl_2 (35 mL) at ambient temperature was added slowly via addition funnel a solution of alcohol **S2** (1.90 g, 11.37 mmol, 1 equiv.) in CH_2Cl_2 (20 mL) and the reaction was monitored by TLC. After 2.5 hours the reaction was filtered through a pad of Celite and concentrated carefully at the rotavap (ATTENTION: aldehyde **7** is volatile). The crude product was purified by FCC (pentane/Et_2O 7:3) to furnish **7** (1.32 g, 71%) as a colorless liquid. NMR data matched those in the literature.[2]
R_f 0.6 (7:3 pentane/Et_2O, CAM stain). ^{1}H-NMR (200 MHz, $CDCl_3$) *δ* 9.73 (t, *J* = 1.5 Hz, 1H), 3.37 (t, *J* = 6.2, 2H), 2.45 (td, *J* = 7.0 and 1.5 Hz, 2H), 1.88-1.64 (m, 4H); ^{13}C NMR (50 MHz, $CDCl_3$) *δ* 201.8, 42.8, 33.1, 31.9, 20.6.

Synthesis of (*S*)-5 following the protocol by Ma and co-workers.[3]

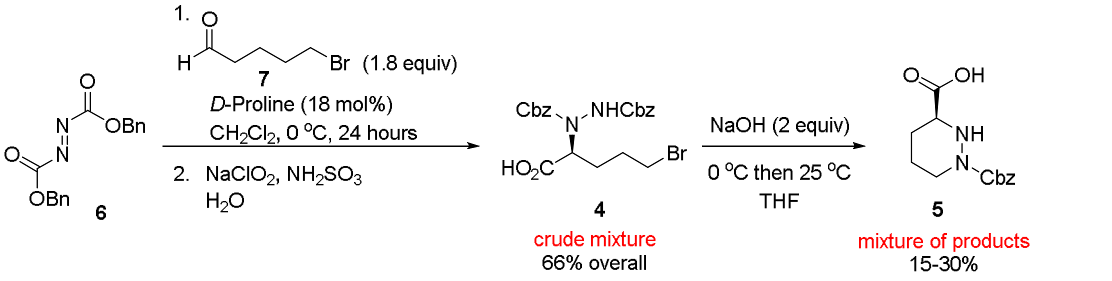

To a stirring solution of aldehyde **7** (1.30 g, 7.87 mmol, 1.8 equiv.) in dry CH_2Cl_2 (11 mL) at 0 °C was added *D*-proline (91 mg, 0.78 mmol, 0.18 equiv.) followed by dibenzylazodicarboxylate (1.30 g, 4.37 mmol, 1 equiv.) and the mixture was left stirring at the same temperature for 15 hours. TLC analysis showed full consumption of the starting material (petroleum ether 40-60 °C /EtOAc 9:1, PMA stain). Sulfamic acid (764 mg, 7.87 mmol, 1.8 equiv.) was added in one portion followed by $NaClO_2$ (1.13 M, 533 mg 80% $NaClO_2$ in 5.2 mL H_2O) slowly while the color of the reaction turns green. After 10 minutes (TLC analysis showed disappearance of the intermediate aldehyde, petroleum ether 40-60 °C /EtOAc 9:1, PMA stain) the reaction was quenched by addition of Na_2SO_3 (aq. saturated) and stirred for 1 hour at room temperature. Then, the mixture was transferred to a separatory funnel and the layers were separated. The aqueous layer was washed with CH_2Cl_2 (2 x 10 mL) and the combined organic layers were washed with brine (30 mL), dried (Na_2SO_4), filtered and concentrated in vacuo. The crude product was purified by FCC (petroleum ether 40-60 °C /EtOAc 1:1 to remove the byproduct and then neat EtOAc) to furnish **4** (1.37 g, 66%) as a white solid.

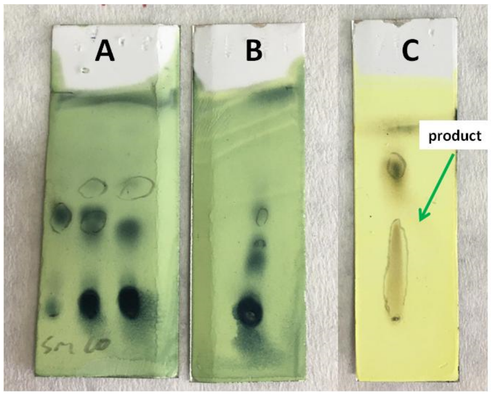

Figure S2. TLC analysis of the reaction sequence. **A**) TLC (petroleum ether 40-60 °C /EtOAc 9:1) of the organocatalytic reaction after 15 hours at 0 °C. In the left lane we have the starting material (sm), in the middle lane the co-spot of sm and reaction mixture (rm), and in the right lane the rm. We can see full consumption of sm. **B**) TLC (petroleum ether 40-60 °C /EtOAc 9:1) after the Pinnick oxidation. **C**) A more polar TLC after the Pinnick oxidation (petroleum ether 40-60 °C /EtOAc 1:1). The product **4** is the lower spot that "tails".

To a stirring solution of acid **4** (1.30 g, 2.71 mmol, 1 equiv.) in THF (9 mL) at 0 °C was added solid NaOH (217 mg, 5.42 mmol, 2 equiv.) and the mixture was stirred at the same temperature for 24 hours. Then, the reaction was allowed to reach ambient temperature and was left stirring for an additional 4 hours. Subsequently, NaOH 1N (aq., 3 mL) and sat. $NaHCO_3$ (aq., 4 mL) were added, the mixture was transferred to a separatory funnel and the layers were separated. The organic layer was washed with sat. $NaHCO_3$ (5 mL) and discarded. The aqueous layer was washed with petroleum ether 40-60 °C (2 x 5 mL) and then was acidified to pH 4-5 with

HCl 37%. The aqueous phase was extracted with EtOAc (3 x 5 mL), dried and concentrated in vacuo to furnish a mixture of bis- and mono-Cbz Piz (307 mg) as colorless glue.

Synthesis of (*R*)-5 following the global deprotection and selective mono-protection sequence.[4]

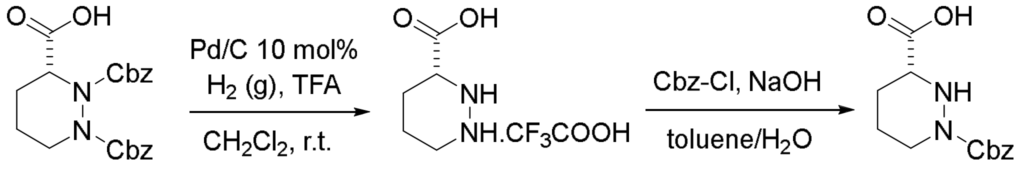

13 **2** **5**

To a stirring solution of acid **13** (1.56 g, 3.92 mmol, 1 equiv.) in dichloromethane (159 mL) was added 10% Pd/C (1.4 g) and trifluoroacetic acid (2.9 mL, 39.2 mmol, 10 equiv). The suspension was stirred under a hydrogen atmosphere (balloon) at 23°C for 12h. The mixture was then filtered through a pad of Celite to remove the catalyst, the filter cake was washed thoroughly with MeOH and the filtrate was concentrated in vacuo to give the trifluroacetic acid salt **2** as a slurry oil. The product was advanced directly to the next step.
^{1}H-NMR (200 MHz, CD_3OD) δ 3.98 (br, 1H), 3.41-3.13 (m, 2H), 2.25-1.91 (m, 4H).[4a]

To a mixture of the above salt **2** (3.92 mmol, 1 equiv.) and NaOH (470 mg, 11.76 mmol, 3.0 equiv.) in water (11.5 mL) was added a solution of benzyl chloroformate (0.56 mL, 3.92 mmol, 1.0 equiv.) in toluene (8 mL) at +10 °C. After being stirred for 15 hours at room temperature, the reaction mixture was transferred to a separatory funnel and the layers were separated. The aqueous layer was washed with Et_2O (10 mL), acidified with 1N HCl to pH 4 and extracted with AcOEt (3 x 15 mL). The combined organic layers were washed with brine, dried (Na_2SO_4) and concentrated in vacuo. The residue was purified by FCC ($CHCl_3$/MeOH: 9/1) to give the mono-protected acid in a 15% yield (150 mg) as yellowish oil.[4b]

Synthesis of (*R*)-13 following the Hamada protocol.[4a]

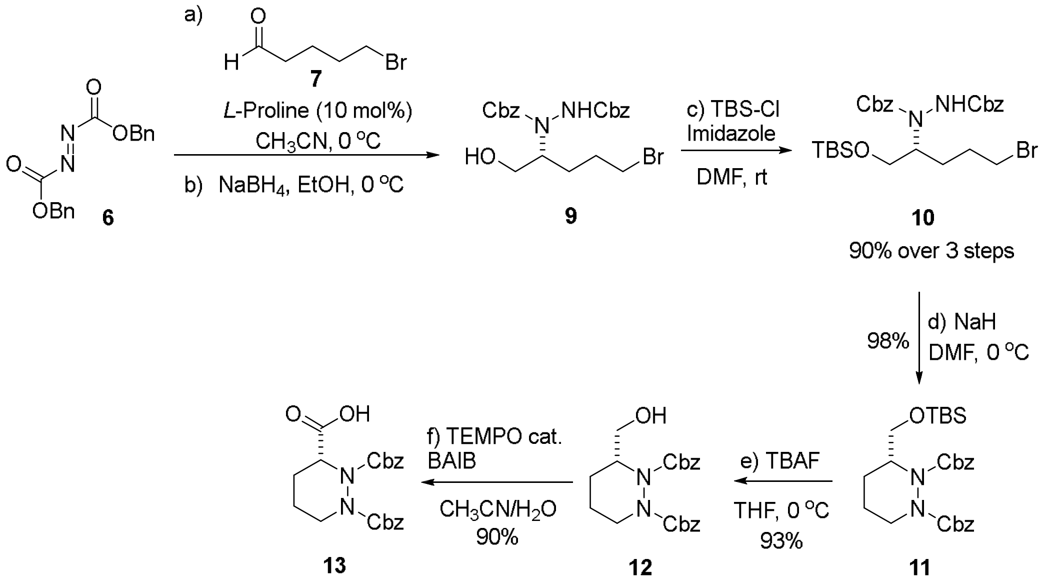

To a stirring solution of aldehyde **7** (1.10 g, 6.67 mmol, 1.5 equiv.) in dry CH_3CN (32 mL) at 0 °C was added dibenzylazodicarboxylate**6** (1.33 g, 4.45 mmol, 1 equiv.) followed by *L*-proline (51 mg, 0.45 mmol, 0.1 equiv.) and the mixture was stirred at the same temperature for 20 hours. Then, $NaBH_4$ (168 mg, 4.45 mmol, 1 equiv.) and EtOH (13 mL) were added sequentially and the reaction was left stirring at 0 °C for an additional 1 hour before it was quenched by slow addition of 5% citric acid (aq., 5-6 mL). After stirring 5 minutes at ambient temperature the reaction was concentrated in vacuo, re-dissolved in EtOAc (30 mL) and transferred to a separatory funnel. The organic phase was washed with brine (30 mL) and the aqueous phase was back-extracted with EtOAc (2 x 20 mL). The combined organic layers were dried (Na_2SO_4), filtered and concentrated. The crude product was purified by FCC (petroleum ether 40-60 °C /EtOAc 6:4) to give alcohol **9** as a white solid.
R_f 0.3 (7:3 petroleum ether 40-60 °C/EtOAc, PMA stain). ^{1}H-NMR (200 MHz, $CDCl_3$) δ 7.32 (br, 10H), 6.53 (br, 1H), 5.17 (br, 4H), 3.56-3.24 (m, 4H), 1.90 (br, 1H), 1.60-1.41 (br, 4H). MS (ESI) *m/z* (%): 487.2 [M+Na, (100)]$^+$, 489.2 [M+Na, (80)]$^+$. The enantiomeric ratio of (*S*)-**9** was determined to be >99% *ee* by chiral HPLC (CHIRALPAK® OD-H, hexane/iPrOH/TFA 90:10:0.1, 0.6 mL/min, 254 nm, 32.4 min).

To a stirring solution of the above alcohol **9** (4.45 mmol, 1 equiv.) in dry DMF (52 mL) was added imidazole (1.51 g, 22.25 mmol, 5 equiv.) followed by TBS-Cl (805 mg, 5.34 mmol, 1.2 equiv.) and the reaction was left stirring at ambient temperature until TLC analysis showed full conversion (approx. 3 hours). Then it was diluted with Et_2O (50 mL) and transferred to a separatory funnel. The organic layer was washed with H_2O (2 x 30 mL) and the aqueous layers were back-extracted with Et_2O (2 x 25 mL). The combined organic phases were then washed with brine (30 mL), dried (Na_2SO_4), filtered and concentrated in vacuo. The crude product was purified by FCC (petroleum ether 40-60 °C /EtOAc 9:1) to furnish bromide **10** (2.31 g, 90% for three steps) as a white solid.
R_f 0.7 (8:2 petroleum ether 40-60 °C /EtOAc, PMA stain). ^{1}H-NMR (200 MHz, $CDCl_3$) δ 7.34-7.23 (br, 10H), 6.57 (br, 1H), 5.14 (br, 4H), 4.29 (br, 1H), 3.67-3.37 (m, 4H), 1.88-1.47 (m, 4H), 0.86 (s, 9H), 0.04 (s, 6H); ^{13}C NMR (50 MHz, $CDCl_3$) δ 157.0, 156.1, 135.7, 128.6, 128.4, 128.2, 67.8, 62.9, 29.0, 26.7, 26.0, 25.7, 18.0, -3.5, -5.5. MS (ESI) *m/z* (%): 581.2 [M+H, (100)]$^+$, 579.4 [M+H, (90)]$^+$.

A stirring solution of bromide **10** (2.30 g, 3.98 mmol, 1 equiv.) in dry DMF (25 mL) under Ar was cooled to 0 °C and NaH (60%, 280 mg, 6.97 mmol, 1.7 equiv.) was added in three portions

over a period of 30 minutes. After stirring at the same temperature for an additional 2 hours TLC analysis showed full conversion. The reaction was quenched by slow addition of 5% citric acid (aq., 10 mL) and then it was allowed to reach room temperature and it was transferred to a separatory funnel. The layers were separated and the aqueous phase was back-extracted with Et_2O (50 mL and then 2 x 25 mL). The combined organic layers were washed with brine (30 mL), dried (Na_2SO_4), filtered and concentrated in vacuo. The crude product was purified by FCC (petroleum ether 40-60 ^oC /EtOAc 9:1) to furnish silyl ether **11** (1.95 g, 98%) as a colourless oil.
R_f 0.5 (85:15 petroleum ether 40-60 ^oC /EtOAc, CAM stain). ^1H-NMR (200 MHz, $CDCl_3$) δ 7.33-7.28 (br, 10H), 5.19-5.13 (m, 4H), 4.44-4.06 (m, 1H), 3.90-3.50 (m, 3H), 3.01 (br, 1H), 1.90-1.67 (br, 3H), 1.59-1.43 (br, 1H), 0.87 (s, 9H), 0.09 (s, 6H); ^{13}C NMR (50 MHz, $CDCl_3$) δ 156.5, 136.2, 136.0, 128.4, 127.8, 67.7, 67.5, 67.3, 60.8, 54.2, 44.3, 25.7, 18.8, 18.1, -5.6. MS (ESI) *m/z* (%): 499.4 $[M+H, (100)]^+$.

A stirring solution of **11** (1.95 g, 3.91 mmol, 1 equiv.) in dry THF (30 mL) under Ar was cooled to 0 ^oC and TBAF (1M in THF, 4.7 mL, 4.69 mmol, 1.2 equiv.) was added. After 1 hour (TLC analysis showed full conversion) the reaction was quenched by the addition brine (20 mL) and the mixture was transferred to a separatory funnel and was extracted with EtOAc (3 x 20 mL). The combined organic layers were dried (Na_2SO_4), filtered and concentrated in vacuo. The crude product was purified by FCC (petroleum ether 40-60 ^oC /EtOAc 1:1) to furnish primary alcohol **12** (1.39 g, 93%) as a colourless oil.
R_f 0.4 (1:1 petroleum ether 40-60 ^oC /EtOAc, CAM stain). ^1H-NMR (200 MHz, $CDCl_3$) δ 7.37 (br, 10H), 5.22 (br, 4H), 4.48 (br, 1H), 4.30-4.06 (m, 1H), 3.81-3.50 (m, 2H), 3.10 (br, 1H), 1.85-1.50 (m, 4H); ^{13}C NMR (101 MHz, $CDCl_3$) δ 156.9, 155.0, 135.9, 135.8, 135.5, 128.8, 128.7, 128.6, 128.5, 128.4, 128.3, 128.1, 127.9, 68.6, 68.5, 68.3, 60.7, 60.1, 19.8, 19.5. MS (ESI) *m/z* (%): 402.1 $[M+NH_4, (100)]^+$, 385.4 $[M+H, (76)]^+$.

To a stirred solution of alcohol **12** (1.63 g, 4.24 mmol, 1 equiv.) in CH_3CN (4.4 mL) and H_2O (4.4 mL) were added TEMPO (133 mg, 0.85 mmol, 0.2 equiv.) and BAIB (3.0 g, 9.33 mmol, 2.2 equiv.) at room temperature. After stirring the mixture at the same temperature for 2 h, water was added and the mixture was extracted with ethyl acetate (3 x 20 mL). The combined organic layers were washed with brine (20 mL), dried (Na_2SO_4), filtered and concentrated in vacuo. The residue was purified by FCC (petroleum ether 40-60 ^oC /EtOAc/formic acid; 1:1:0.01) to furnish **13** (1.57 g, 93%) as a colorless glue.
R_f 0.4 (9:1 $CHCl_3$/MeOH, PMA stain). For (*S*)-**13**: $[\alpha]_D^{23}$= -17 (*c* 1, $CHCl_3$), {Lit.[5]$[\alpha]_D^{23}$ = -19.6 (*c* 1, $CHCl_3$)};^1H-NMR (200 MHz, $CDCl_3$) δ 8.86 (br, 1H), 7.35-7.24 (br s, 10H), 5.31-4.97 (m, 5H), 4.30-3.97 (br, 1H), 3.29-2.98 (br, 1H), 2.33-1.60 (m, 4H); ^{13}C NMR (101 MHz, $CDCl_3$) δ 177.1, 171.2, 137.6, 135.1, 130.4, 128.8, 128.6, 128.3, 128.0, 127.6, 94.5, 69.4, 69.3, 68.9, 68.3, 20.8, 20.5, 20.1. MS (ESI) *m/z* (%): 397.0 $[M-H, (100)]^-$.

Determination of enantiopurity of 1-((Benzyloxy)carbonyl)hexahydropyridazine-3-carboxylic acid (5)

The enantiomeric ratio of compound **5** was determined after derivatization to the corresponding allyl ester (**S3**).

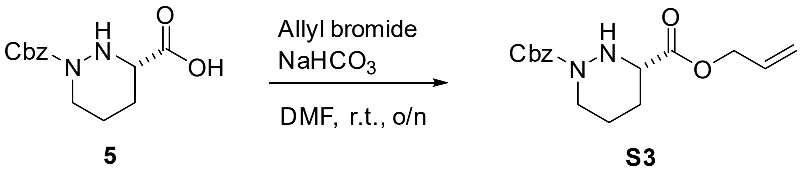

To a stirring solution of acid **5** (80 mg, 0.30 mmol, 1 equiv.) in dry DMF (1.6 mL) was added sequentially $NaHCO_3$ (102 mg, 1.20 mmol, 4.0 equiv.) and allyl bromide (50 µL, 0.61 mmol, 2.0 equiv.) and the mixture was stirred at ambient temperature overnight. The reaction was quenched by addition of H_2O (2 mL) and the product was extracted with Et_2O (3 x 5 mL). The combined organic layers were washed with brine (5 mL), dried (Na_2SO_4), filtered and concentrated in vacuo. The residue was purified by FCC (petroleum ether 40-60 °C /Et_2O 1:1) to furnish the desired allyl ester **S3** as a colorless liquid.

R_f 0.2 (1:1 petroleum ether 40-60 °C /Et_2O, PMA stain). For (*S*)-**S3**: $[\alpha]_D^{23}$= -26 (*c* 1, $CHCl_3$), {Lit.[6]$[\alpha]_D^{23}$ = -30.2 (*c* 1, $CHCl_3$)};^{1}H-NMR (200 MHz, $CDCl_3$) δ 7.39-7.26 (m, 5H), 5.99-5.80 (m, 1H), 5.36-5.17 (m, 4H), 4.61 (dt, *J* = 5.8 and 1.4 Hz, 2H), 4.07-3.90 (m, 1H), 3.63-3.48 (m, 1H), 3.25-3.00 (m, 1H), 2.17-2.00 (m, 1H), 1.85-1.46 (m, 3H). MS (ESI) *m/z* (%): 305.2 $[M+H, (100)]^+$. The enantiomeric ratio of (*S*)-**S3** was determined to be 94:6 (88% *ee*) by chiral HPLC (CHIRALPAK ® AD-H, hexane/iPrOH 90:10, 1.0 mL/min, 230 nm, 17.6 minor and 23.4 major).

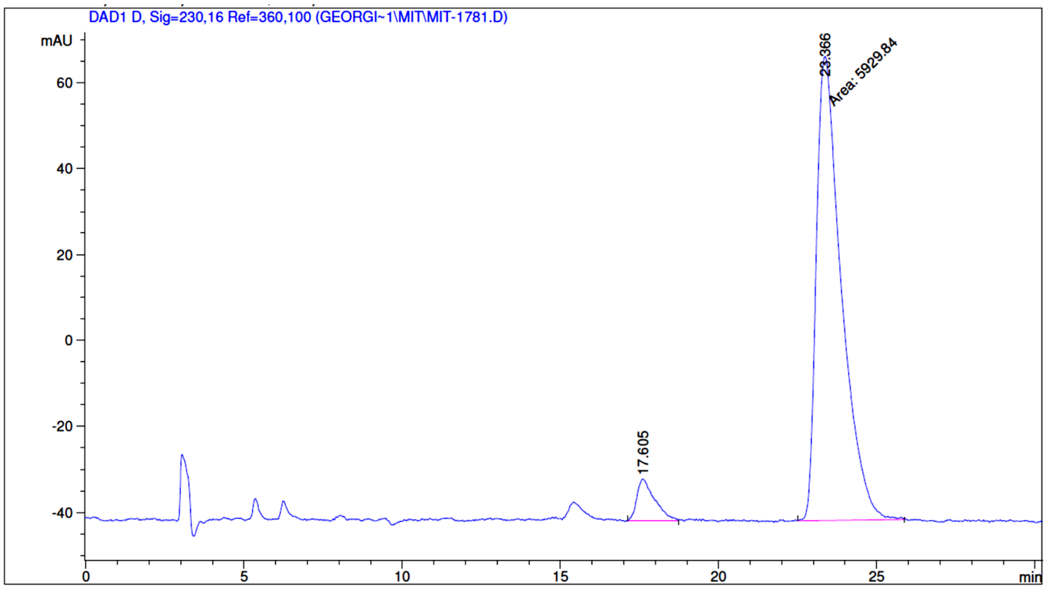

Area Percent Report

Peak #	RetTime [min]	Type	Width [min]	Area [mAU*s]	Height [mAU]	Area %
1	17.605	VV	0.4761	386.80084	9.80773	6.1235
2	23.366	MM	0.9159	5929.84375	107.90417	93.8765
Totals :				6316.64459	117.71189	

For (*R*)-**S3**

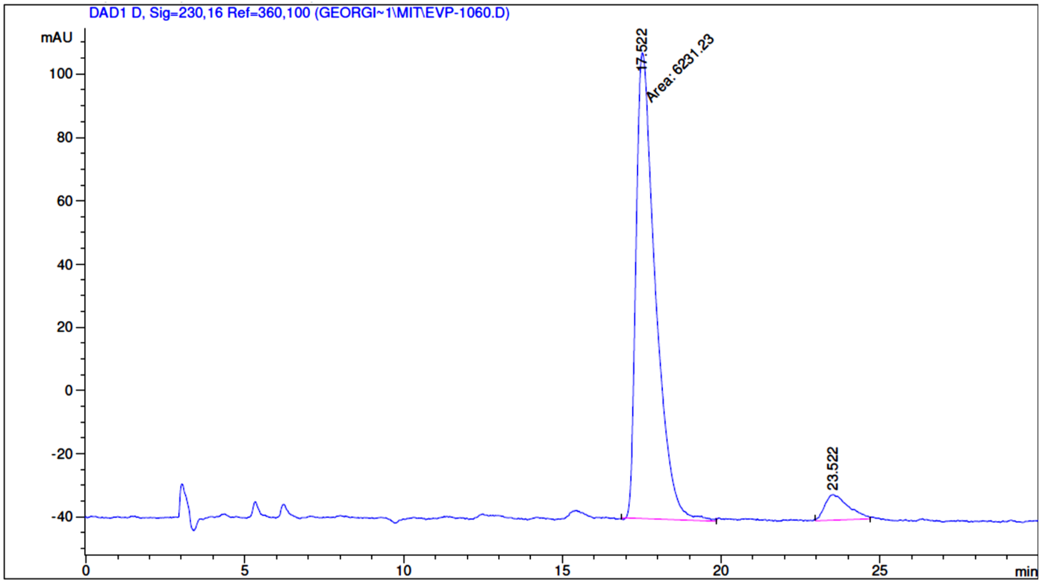

Area Percent Report

Peak #	RetTime [min]	Type	Width [min]	Area [mAU*s]	Height [mAU]	Area %
1	17.522	MM	0.7056	6231.22852	147.17540	93.7418
2	23.522	VV	0.6180	415.99628	8.00828	6.2582
Totals :				6647.22479	155.18368	

Racemic **S3** was prepared by mixing equal amounts of chiral (*S*)- and (*R*)-**S3**

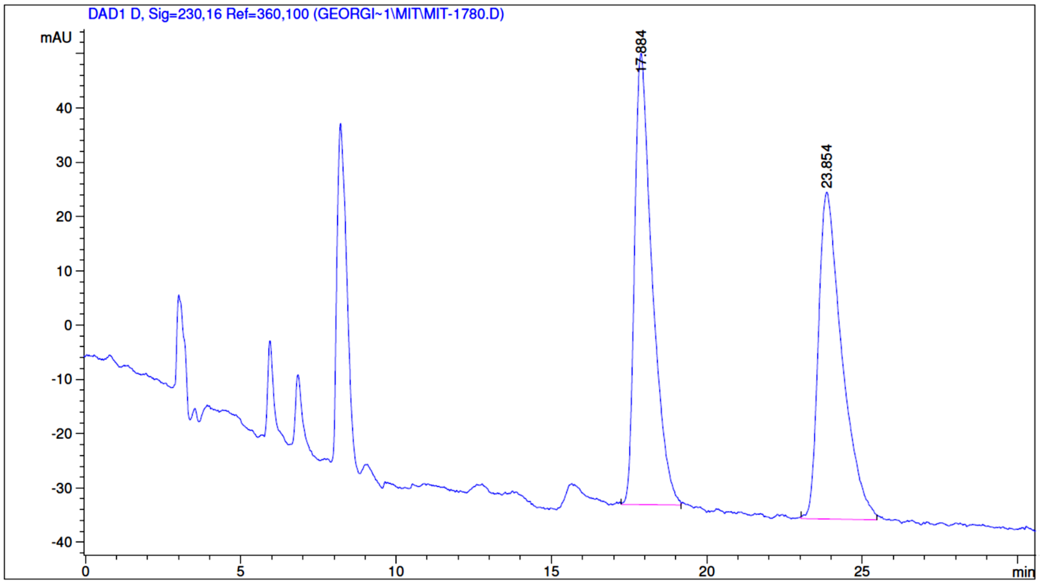

Area Percent Report

Peak #	RetTime [min]	Type	Width [min]	Area [mAU*s]	Height [mAU]	Area %
1	17.884	VV	0.5408	3279.98193	83.04240	50.8530
2	23.854	BB	0.6384	3169.95142	60.19385	49.1470
Totals :				6449.93335	143.23626	

Spectra

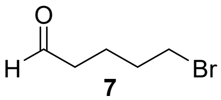
O
H
Br

7

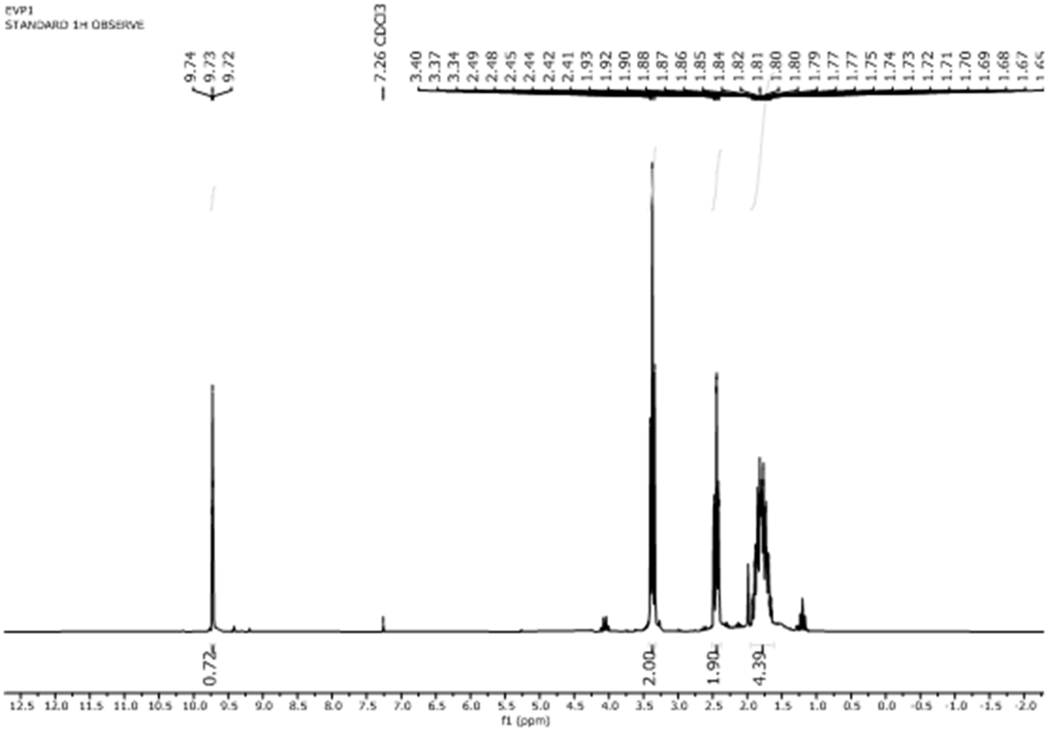
EVP1
STANDARD 1H OBSERVE
9.74
9.73
9.72
7.26 CDCl3
3.40
3.37
3.34
2.49
2.48
2.45
2.44
2.42
2.41
1.93
1.92
1.90
1.88
1.87
1.86
1.85
1.84
1.82
1.81
1.80
1.79
1.77
1.77
1.75
1.74
1.73
1.72
1.71
1.70
1.69
1.68
1.67
0.72
2.00
1.90
4.39
f1 (ppm)

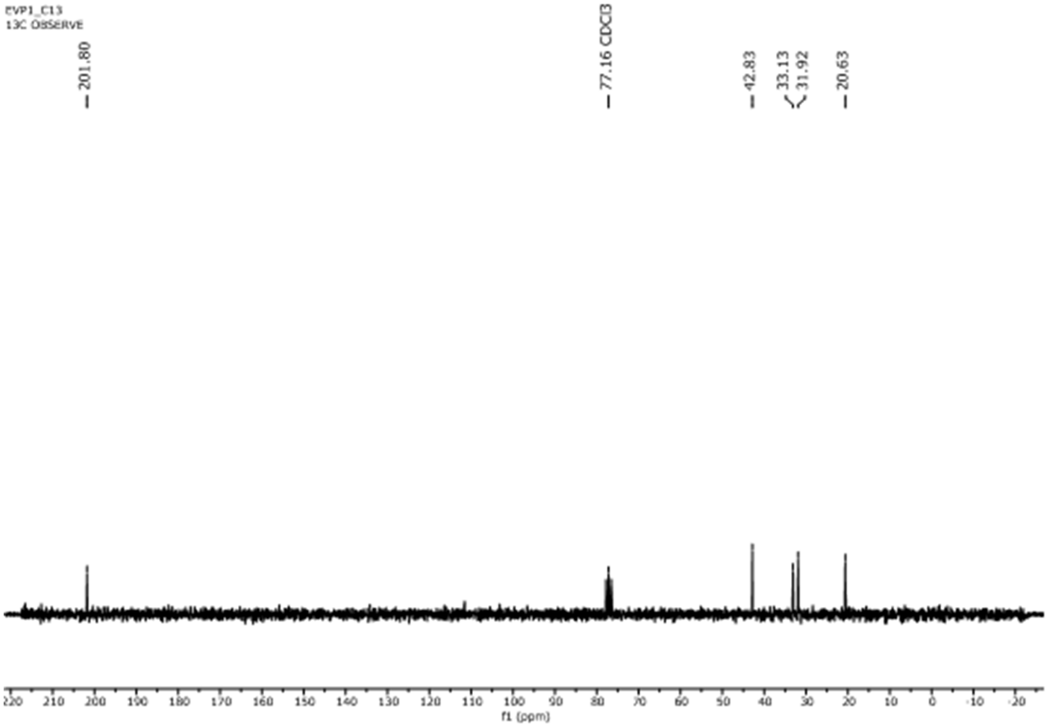
EVP1_C13
13C OBSERVE
201.80
77.16 CDCl3
42.83
33.13
31.92
20.63
f1 (ppm)

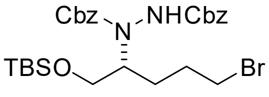
Cbz
NHCbz
N
TBSO
Br

10

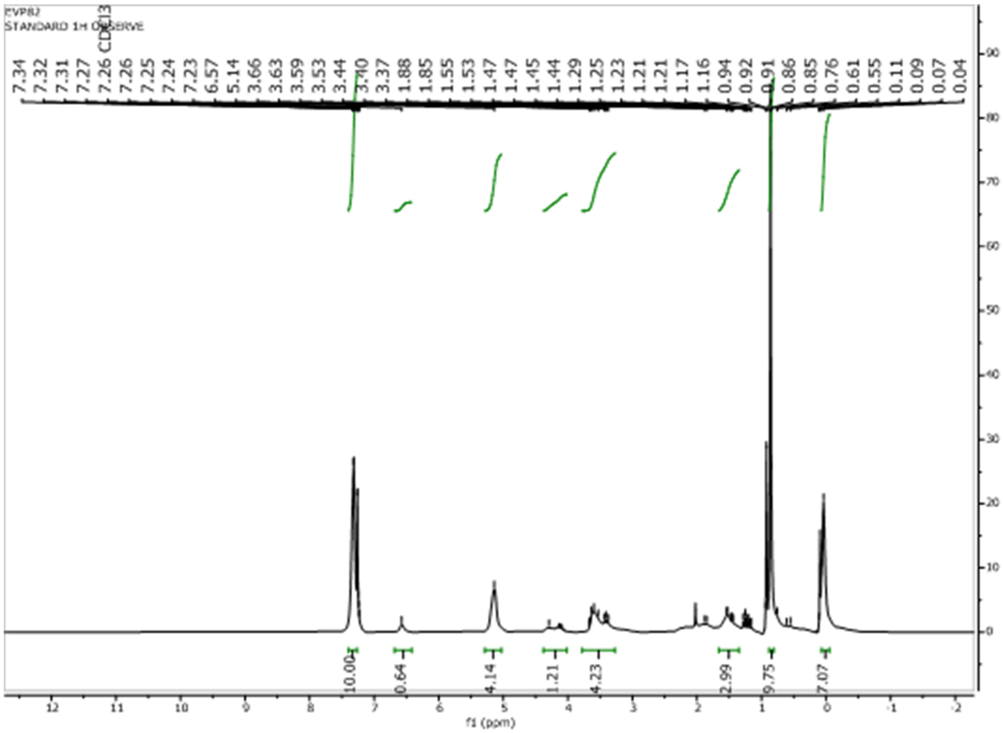
EVP82
STANDARD 1H OBSERVE
CDCl3
7.34 7.32 7.31 7.27 7.26 7.26 7.25 7.24 7.23 6.57 5.14 3.66 3.63 3.59 3.53 3.44 3.40 3.37 1.88 1.85 1.55 1.53 1.47 1.47 1.45 1.44 1.29 1.25 1.23 1.21 1.21 1.17 1.16 0.94 0.92 0.91 0.86 0.85 0.76 0.61 0.55 0.11 0.09 0.07 0.04
10.00
0.64
4.14
1.21
4.23
2.99
9.75
7.07
f1 (ppm)

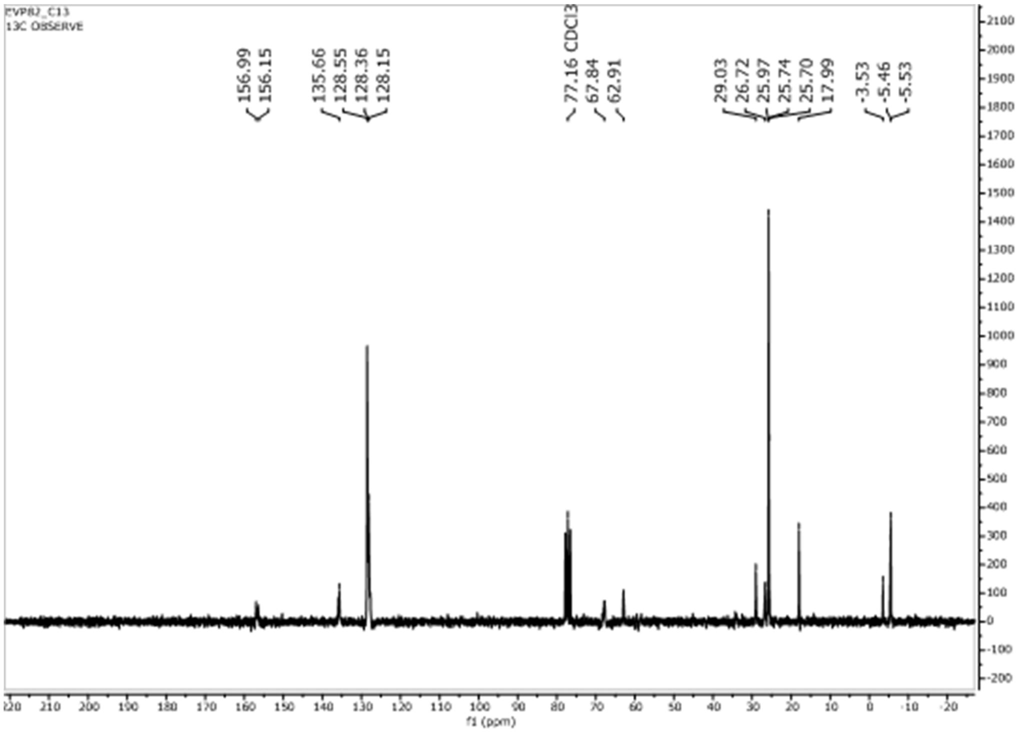
EVP82_C13
13C OBSERVE
156.99
156.15
135.66
128.55
128.36
128.15
77.16 CDCl3
67.84
62.91
29.03
26.72
25.97
25.74
25.70
17.99
-3.53
-5.46
-5.53
f1 (ppm)

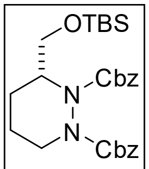

OTBS
N
Cbz
N
Cbz

11

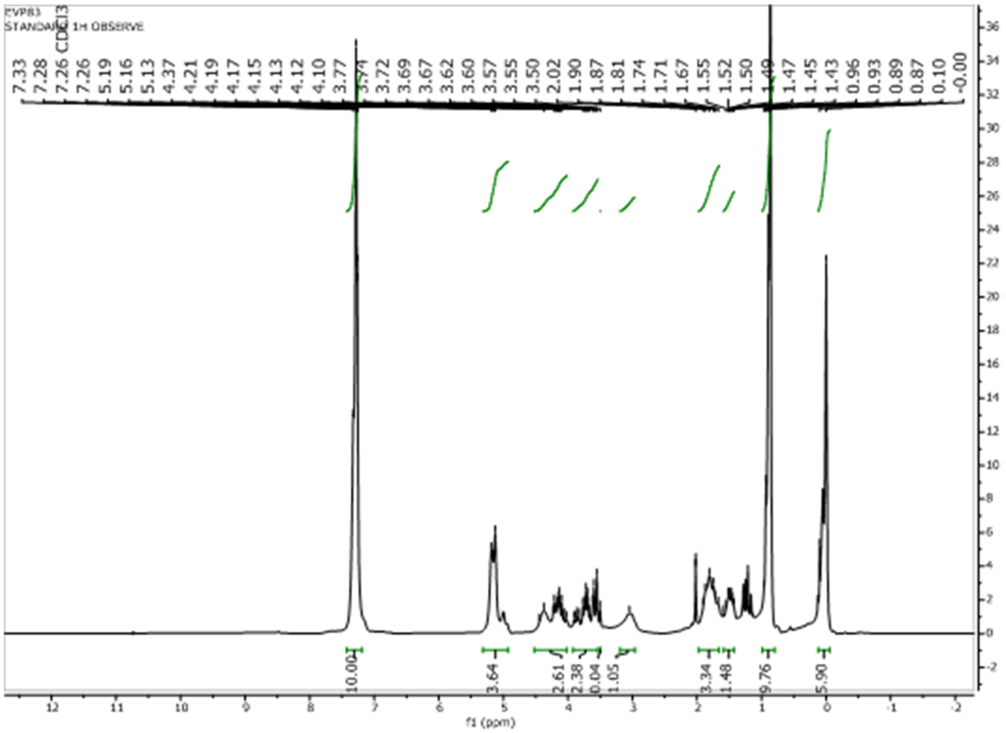

EVP83
STANDARD 1H OBSERVE
CDCl3
10.00
3.64
2.61
2.38
0.04
1.05
3.34
1.48
9.76
5.90
f1 (ppm)

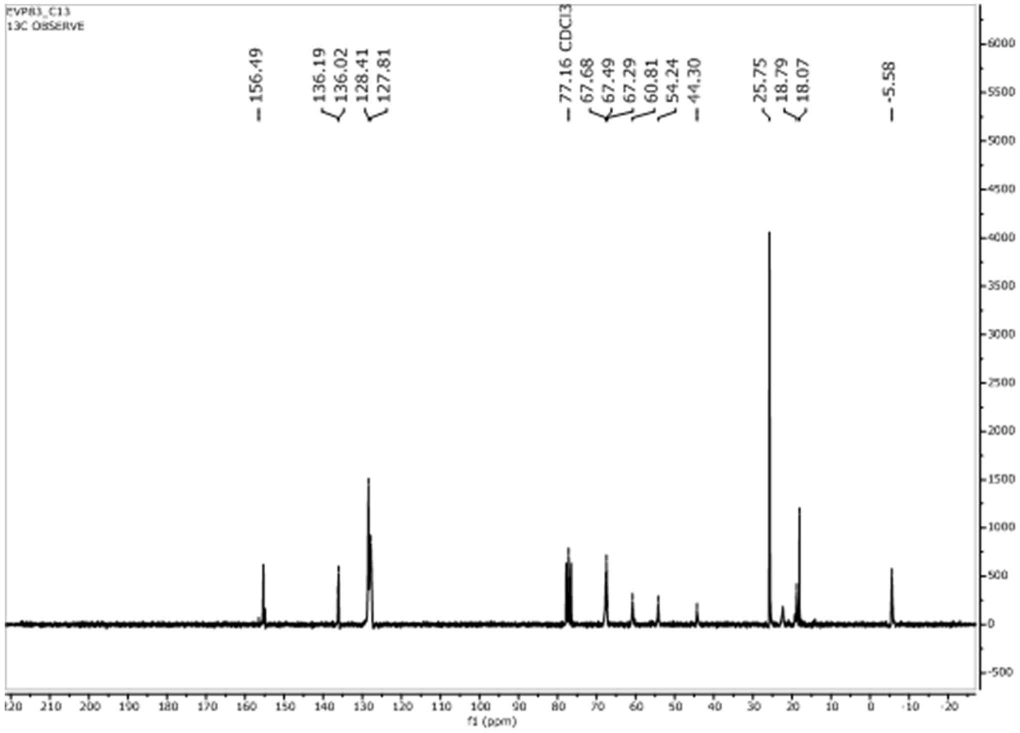

EVP83_C13
13C OBSERVE
156.49
136.19
136.02
128.41
127.81
77.16 CDCl3
67.68
67.49
67.29
60.81
54.24
44.30
25.75
18.79
18.07
-5.58
f1 (ppm)

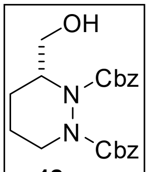
OH
N
Cbz
N
Cbz

12

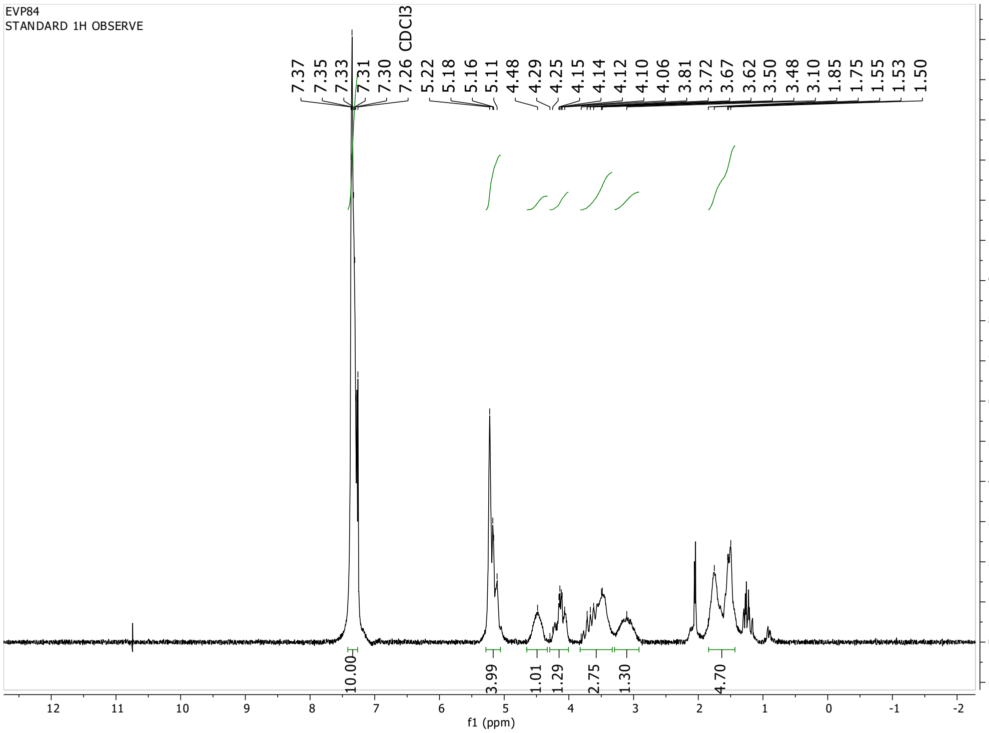
EVP84
STANDARD 1H OBSERVE
7.37
7.35
7.33
7.31
7.30
7.26 CDCl3
5.22
5.18
5.16
5.11
4.48
4.29
4.25
4.15
4.14
4.12
4.10
4.06
3.81
3.72
3.67
3.62
3.50
3.48
3.10
1.85
1.75
1.55
1.53
1.50
10.00
3.99
1.01
1.29
2.75
1.30
4.70
f1 (ppm)

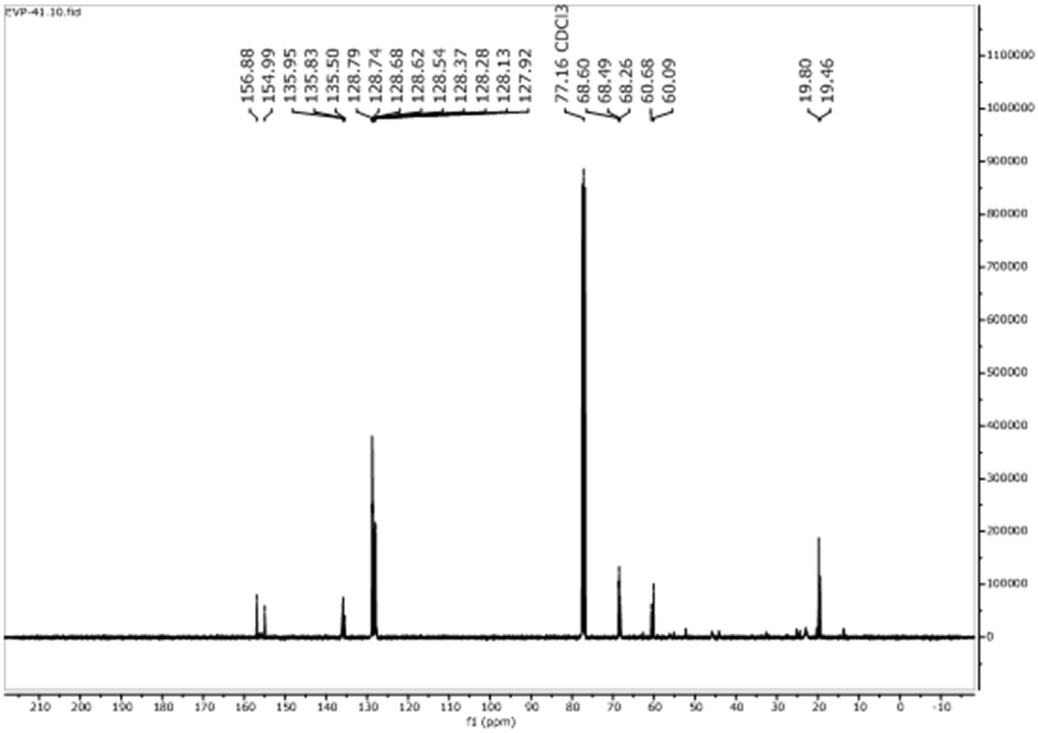
EVP-41.10.fid
156.88
154.99
135.95
135.83
135.50
128.79
128.74
128.68
128.62
128.54
128.37
128.28
128.13
127.92
77.16 CDCl3
68.60
68.49
68.26
60.68
60.09
19.80
19.46
f1 (ppm)

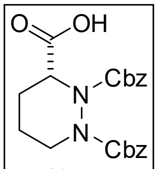

O
OH
N
Cbz
N
Cbz

13

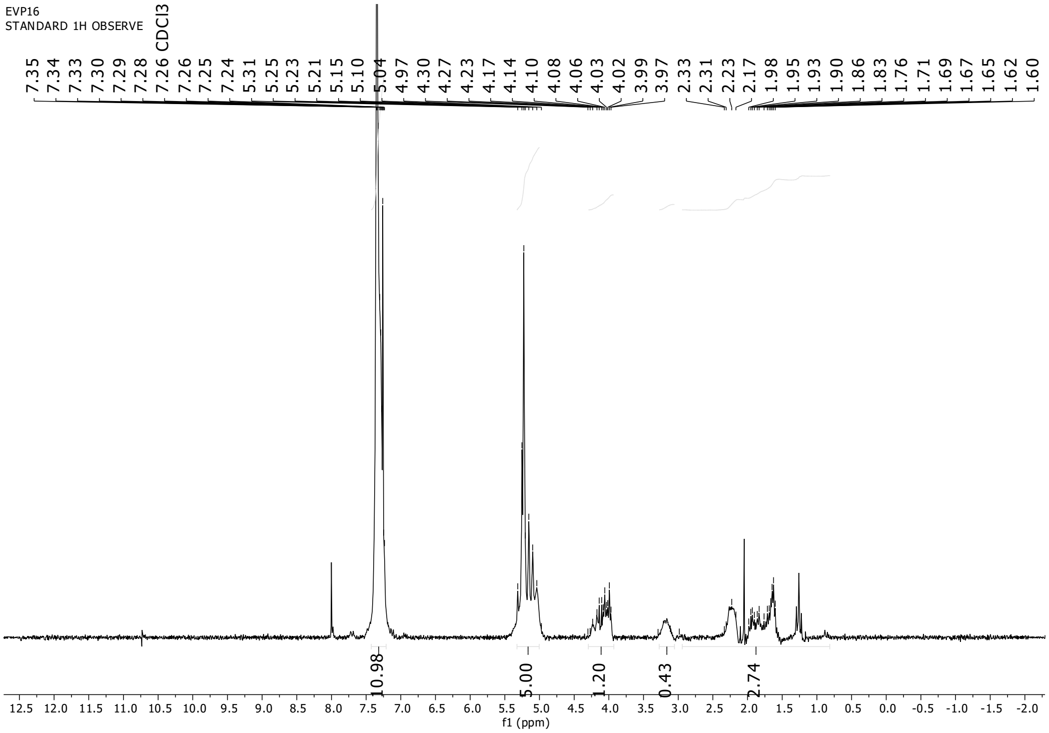

EVP16
STANDARD 1H OBSERVE
7.35 7.34 7.33 7.30 7.29 7.28 7.26 CDCl3 7.26 7.25 7.24 5.31 5.25 5.23 5.21 5.15 5.10 5.04 4.97 4.30 4.27 4.23 4.17 4.14 4.10 4.08 4.06 4.03 4.02 3.99 3.97 2.33 2.31 2.23 2.17 1.98 1.95 1.93 1.90 1.86 1.83 1.76 1.71 1.69 1.67 1.65 1.62 1.60
10.98
5.00
1.20
0.43
2.74
12.5 12.0 11.5 11.0 10.5 10.0 9.5 9.0 8.5 8.0 7.5 7.0 6.5 6.0 5.5 5.0 4.5 4.0 3.5 3.0 2.5 2.0 1.5 1.0 0.5 0.0 -0.5 -1.0 -1.5 -2.0
f1 (ppm)

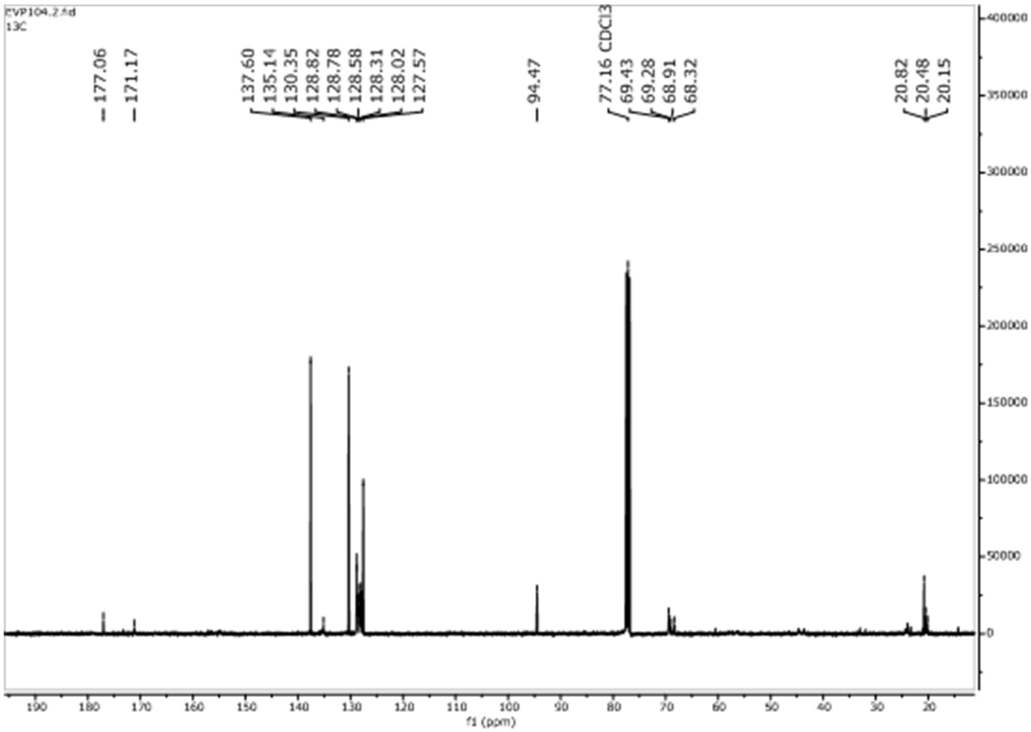

EVP104.2.fid
13C
177.06
171.17
137.60 135.14 130.35 128.82 128.78 128.58 128.31 128.02 127.57
94.47
77.16 CDCl3
69.43 69.28 68.91 68.32
20.82 20.48 20.15
400000
350000
300000
250000
200000
150000
100000
50000
0
190 180 170 160 150 140 130 120 110 100 90 80 70 60 50 40 30 20
f1 (ppm)

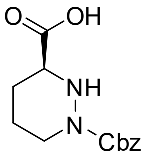
O
OH
NH
N
Cbz

5

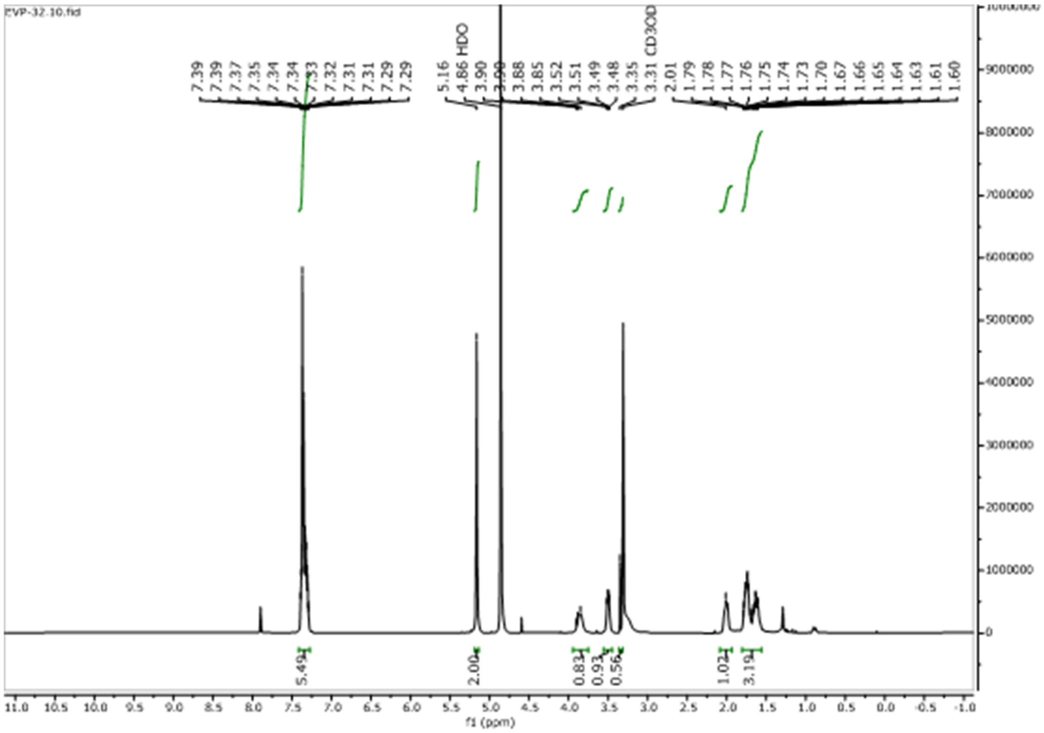
EVP-32.10.fid
7.39
7.39
7.37
7.35
7.34
7.34
7.33
7.32
7.31
7.31
7.29
7.29
5.16
4.86 HDO
3.90
3.88
3.85
3.52
3.51
3.49
3.48
3.35
3.31 CD3OD
2.01
1.79
1.78
1.77
1.76
1.75
1.74
1.73
1.70
1.67
1.66
1.65
1.64
1.63
1.61
1.60
5.49
2.00
0.83
0.93
0.56
1.02
3.19
f1 (ppm)

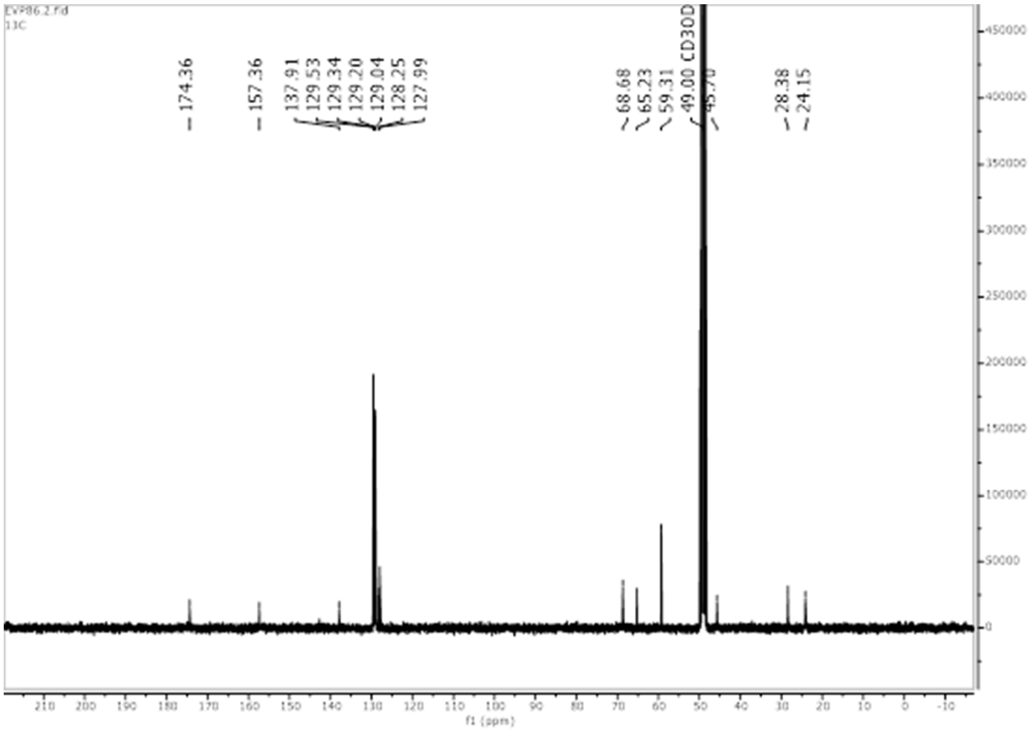
EVP86.2.fid
13C
174.36
157.36
137.91
129.53
129.34
129.20
129.04
128.25
127.99
68.68
65.23
59.31
49.00 CD3OD
45.70
28.38
24.15
f1 (ppm)

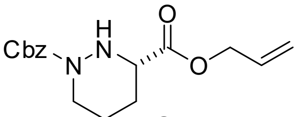

S3

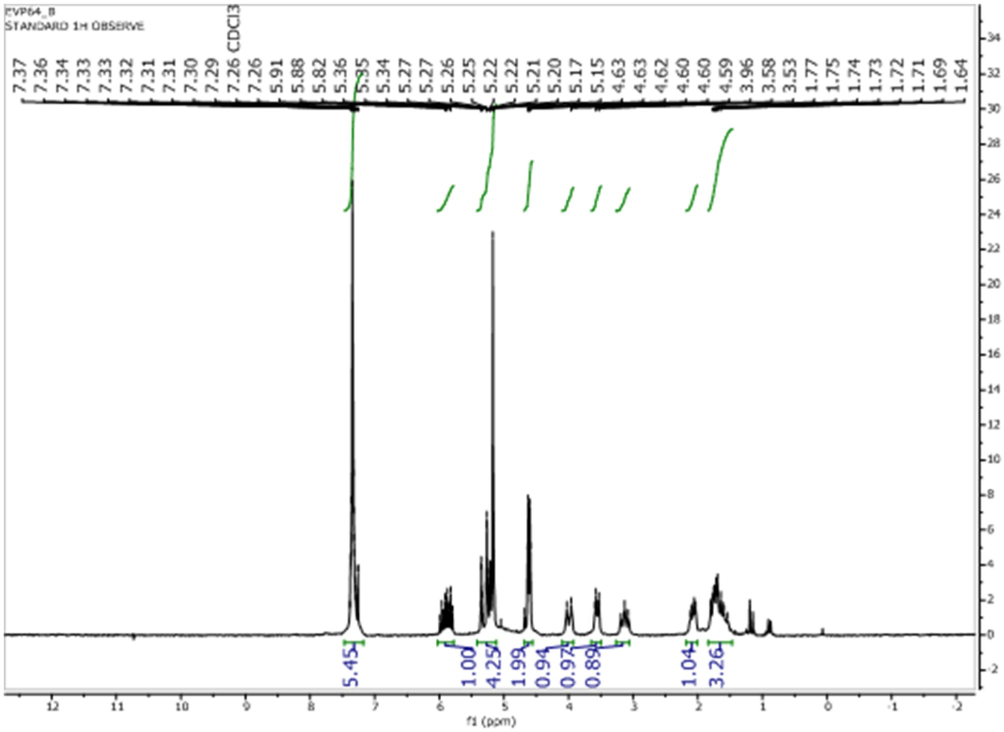

---

[1] (a) For the mono-bromination procedure see: Chong, J. M.; Heuft, M. A.; Rabbat, P. *J. Org. Chem.* **2000**, *65*, 5837-5838. (b) For product characterization see: Thomson, A.; O'Connor, S.; Knuckley, B.; Causey, C. P. *Bioorg. Med. Chem.* **2014**, *22*, 4602-4608.

[2] For product characterization see: Ponath, S.; Menger, M.; Grothues, L.; Weber, M.; Lentz, D.; Strohmann, C.; Christmann, M. *Angew. Chem. Int. Ed.* **2018**, *57*, 11683-11687.

[3] Chen, Y.; Lu, Y.; Zou, Q.; Chen, H.; Ma, D. *Org. Proc. Res. Dev.* **2013**, *17*, 1209-1213.

[4] (a) For the hydrogenolysis step see: Henmi, Y.; Makino, K.; Yoshitomi, Y.; Hara, O.; Hamada, Y. *Tetrahedron: Asymmetry*, **2004**, *15*, 3477-3481. (b) For the selective protection step see: Adams, C. E.; Aguilar, D.; Hertel, S.; Knight, W. H.; Paterson, J. *Synth. Commun.* **1988**, *18*, 2225-2231.

[5] Makino, K.; Henmi, Y.; Terasawa, M.; Hara, O.; Hamada, Y. *Tetrahedron Lett.* **2005**, *46*, 555-558.

[6] Shibahara, S.; Matsubara, T.; Takahashi, K.; Ishihara, J.; Hatakeyama, S. *Org. Lett.* **2011**, *13*, 4700-4703.
